# A longitudinal systems immunologic investigation of acute Zika virus infection in an individual infected while traveling to Caracas, Venezuela

**DOI:** 10.1371/journal.pntd.0007053

**Published:** 2018-12-31

**Authors:** Aaron F. Carlin, Jinsheng Wen, Edward A. Vizcarra, Melanie McCauley, Antoine Chaillon, Kevan Akrami, Cheryl Kim, Annie Elong Ngono, Maria Luz Lara-Marquez, Davey M. Smith, Christopher K. Glass, Robert T. Schooley, Christopher Benner, Sujan Shresta

**Affiliations:** 1 Department of Medicine, School of Medicine, University of California, San Diego, La Jolla, California, United States of America; 2 Division of Inflammation Biology, La Jolla Institute for Immunology, La Jolla, California, United States of America; 3 Veterans Affairs San Diego Healthcare System, San Diego, California, United States of America; 4 Department of Cellular and Molecular Medicine, School of Medicine, University of California, San Diego, La Jolla, California, United States of America; Oregon Health and Science University, UNITED STATES

## Abstract

Zika virus (ZIKV) is an emerging mosquito-borne flavivirus linked to devastating neurologic diseases. Immune responses to flaviviruses may be pathogenic or protective. Our understanding of human immune responses to ZIKV in vivo remains limited. Therefore, we performed a longitudinal molecular and phenotypic characterization of innate and adaptive immune responses during an acute ZIKV infection. We found that innate immune transcriptional and genomic responses were both cell type- and time-dependent. While interferon stimulated gene induction was common to all innate immune cells, the upregulation of important inflammatory cytokine genes was primarily limited to monocyte subsets. Additionally, genomic analysis revealed substantial chromatin remodeling at sites containing cell-type specific transcription factor binding motifs that may explain the observed changes in gene expression. In this dengue virus-experienced individual, adaptive immune responses were rapidly mobilized with T cell transcriptional activity and ZIKV neutralizing antibody responses peaking 6 days after the onset of symptoms. Collectively this study characterizes the development and resolution of an *in vivo* human immune response to acute ZIKV infection in an individual with pre-existing flavivirus immunity.

## Author summary

Zika virus (ZIKV) is an emerging flaviviral infection that causes significant clinical disease. It is estimated that approximately one half of the world’s population is at risk for ZIKV infection. There are only a limited number of studies describing the human immune response to ZIKV infection. *Carlin et al*. combined conventional and genomic approaches to longitudinally analyze the innate and adaptive immune responses to acute ZIKV infection and its resolution in a person who was infected while traveling in Venezuela during the 2016 ZIKV epidemic year. Genome-wide sequencing in individual cell types revealed that although many populations respond to interferon stimulation, only specific cell populations within peripheral blood mononuclear cells upregulate important inflammatory cytokine gene expression. Additionally, analysis of open chromatin using ATAC-seq suggests that chromatin remodeling at sites containing cell-type specific transcription factor binding motifs may help us understand changes in gene expression. Consistent with previous reports, this individual with prior exposure to dengue virus (DENV), rapidly developed neutralizing anti-ZIKV responses that were cross-reactive with multiple DENV serotypes. Collectively this study combines traditional and genomic approaches to characterize the cell-type specific development of an *in vivo* human immune response to acute ZIKV infection.

## Introduction

Zika virus (ZIKV) is an emerging arthropod-borne flavivirus. It is primarily transmitted by *Aedes sp*. mosquitos but can also be transmitted person to person vertically from mother to child, sexually and in blood during transfusions [1]. Clinical manifestations occur in approximately 20% of infections and can include an acute onset low grade fever, pruritic erythematous macular papular rash, arthralgias and conjunctivitis [2]. Clinically these symptoms can be confused with dengue virus (DENV) or chikungunya virus (CHIKV) infections that are transmitted by the same mosquito vectors and can co-circulate with ZIKV [3]. During pregnancy, ZIKV can cause congenital Zika syndrome and other severe birth defects in fetuses [2]. In adults, ZIKV is associated with life-threatening Guillain-Barré Syndrome (GBS) [4, 5]. The details of how ZIKV bypasses immune restriction to cause disease are still under investigation.

The relationship between flaviviruses and the immune system is complex [6]. On one hand, the immune system can exacerbate viral pathogenesis. For example, ZIKV, like DENV and West Nile virus (WNV), infect innate immune white blood cells early in infection [7–11]. Studies in ZIKV infected children identified monocytes, in particular CD14^+^CD16^+^ intermediate monocytes, and myeloid dendritic cells as the main targets of ZIKV infection in peripheral blood mononuclear cells (PBMCs) [9]. These infected cells may act like a “Trojan horse” to increase spread of the virus to different tissue compartments. Antibody (Ab) responses to flaviviruses are often cross-reactive and have the potential to mediate antibody-dependent enhancement (ADE). While there is no evidence that ADE alters ZIKV pathogenesis in humans, in a mouse model of ZIKV infection, administration of DENV or WNV convalescent plasma increased ZIKV morbidity and mortality through ADE [12]. On the other hand, the development of protective adaptive immune responses is thought to be critical to clear ZIKV infection [6]. Therefore, increasing our understanding of human immune responses to ZIKV infection can lead to better understanding of ZIKV clinical manifestations and pathogenesis and inform the development of vaccines.

Only a small number of studies have examined human responses to ZIKV infection *in vivo*. Analysis of serum inflammatory markers during acute ZIKV infection identified some potential biomarkers associated with neurologic complications [13] and viremia plus moderate symptoms [14]. Monoclonal Abs isolated from four donors infected with ZIKV demonstrated that neutralizing Abs primarily recognized the envelope protein domain III of ZIKV and that Abs recognizing different ZIKV epitopes could alternatively protect against ZIKV challenge or enhance subsequent DENV infection in mice [15]. Another study tracking the development of Ab responses to ZIKV in three DENV-experienced and one DENV-naïve individual found that acute-phase Abs developing during ZIKV infection in DENV-experienced individuals were highly cross-reactive but poorly neutralizing [16]. In a single flavivirus naïve individual, anti-ZIKV B-cell plasma neutralization activity and T-cell responses peaked later between day 15 and day 21 [17]. A large study examining T cell responses to ZIKV in DENV-naïve and DENV-immune patients revealed that DENV exposure prior to ZIKV infection influences the timing, magnitude, and quality of the T cell response [18]. In another study that examined both innate and adaptive immune responses in 5 individuals infected with ZIKV, Lai et. al. observed that flavivirus-experienced individuals developed rapid cross-reactive antibody responses against both DENV and ZIKV as well as activated CD8+ T cell responses, albeit few ZIKV-specific CD8+ T cells were identified [19].

These studies provide insight into human ZIKV infection, but our understanding remains limited due to the small number of reported cases. Additionally, published reports have utilized conventional approaches to study the *in vivo* immune responses to ZIKV. Combining these approaches with genome-wide next-generation sequencing (NGS) analyses could bring new insight into human ZIKV responses and inform direction and design of future studies of immune responses during infection in larger cohorts. As a step towards improving our understanding of human immune responses to acute ZIKV infection through new approaches, we present a detailed immunologic characterization of the innate and adaptive temporal and cell type-specific responses to an acute ZIKV infection in a DENV-experienced patient.

## Methods

### Ethics statement

This research study was approved by the UCSD IRB with Human Research Protections Program # 161060. Written informed consent was obtained from the adult human subject described in this report.

### Sample Collection

After obtaining written informed consent, blood was collected on five occasions d3, d6, d17, d48, and d240 post-onset of symptoms (POS). Urine was collected on d3 and d6 only. Serum was isolated by collecting blood into a plain tube containing no anticoagulant, allowed to clot at room temperature for 20 minutes followed by centrifugation at 1500xg for 10 minutes in a refrigerated centrifuge. Serum was frozen in single use aliquots at -80°C. Peripheral blood mononuclear cells (PBMCs) were isolated from heparinized blood using Histopaque-1077 per manufacturer's instructions and subjected to flow activated cell sorting (FACS) or cryopreserved in 5 million cell aliquots in 90% FBS + 10% DMSO (Hybri-max Sigma) using a Nalgene Mr. Frosty at -80°C for 24 hours before transfer to liquid nitrogen. Cryopreserved cells were thawed rapidly to 37°C and slowly diluted with pre-warmed growth media, followed by gentle pelleting and resuspension in cold FACS staining buffer.

### Virus isolation

Five microliters of d3 POS serum or blood was inoculated into a T25 flask of C6/36 mosquito (*Aedes albopictus*) cells. Supernatants (5 mL) were harvested seven days after culture and titrated via BHK-21 cell-based focus forming assay (FFA) and anti-Flavivirus envelope (E) protein antibody clone 4G2. The urine culture supernatant had a titer of 2.0 x 10^4^ focus forming units (FFU)/mL. Infectious virus in the serum culture supernatant was undetectable.

### Viral sequencing and phylogenetic analysis

Viral RNA from 0.2ml of C6/36 supernatant that was inoculated with d3 POS urine was extracted using the Roche High Pure Viral RNA Kit (Roche) and reversed transcribed using a primer specific method for Zika^Br^ (Forward primer AGTGGAGACGATTGYTGTNGT, Reverse primer AACATGTCTTCTGTGGTCATCCA) (SuperScript III First-Strand Synthesis System for RT-PCR, Invitrogen). cDNA was amplified using Taq polymerase (Roche), cleaned using QIAquick PCR Purification Kit (Qiagen) and sequenced using BDT v3.1 on the ABI 3130xl Genetic Analyzer. Forward and reverse sequences were used to make a contig and manually edited using Bioedit [ref http://www.mbio.ncsu.edu/BioEdit/bioedit.html]. The Basic Alignment Search Tool (BLAST) [ref:] was then used with the resultant sequence [ref: https://blast.ncbi.nlm.nih.gov/Blast.cgi?PROGRAM=blastn&PAGE_TYPE=BlastSearch&LINK_LOC=blasthome] which most closely aligned with other ZIKV NS5 sequences. For phylogenetic analyses, RNA from ZIKV SD001 infected primary human macrophages were aligned to the human hg19 genome using STAR [PMID: 23104886]. Any unmapped reads were used as input for strand-specific *de novo* transcriptome assembly with Trinity [PMID: 21572440]. The longest assembled transcripts were approximately 9 kb, and corresponded to near full-length viral genomes. The resulting alignment from ZIKV SD001 and 435 publicly available ZIKV sequences from NCBI viral genomes resource [20] were used to perform an approximate maximum likelihood phylogenetic tree with PhyML [21]. The tree was rooted with ZIKV (GenBank accession number KY241712) isolated in Asia.

### Flow cytometry

For innate immune cell sorting ten million PBMCs were stained with antibodies against CD3 PE-Cy7, CD19 PE-Cy7 CD20 PE-Cy7, HLADR BV421, CD11c AF700, CD123 PE, CD14 AF488, CD16 APC, CD56 APC-Cy7, and Zombie Aqua Fixable viability dye and separated as shown. For T cell sorting, five million cryopreserved PBMCs were stained with CD16 BV510, CD56 BV510, CD4 APC-eFluor780, CD3 AF700, CD8 BV785, CD45RA BV570, CCR7 PE-Cy7, CXCR5 BV421, CXCR3 BV605, TCR V_24-J_18 BV711, CD226 BB515, CCR6 PerCP-Cy5.5, CCR4 PE, CD25 PE-Dazzle 594, and CD127 AF647 and sorted into CD3^+^ T cell CD4^+^ and CD8^+^ populations. T cells were further analyzed for effector or memory phenotypes, CD4 T helper (Th) subsets based on the expression of chemokine receptors (Th1: CCR6^-^CCR4^-^CXCR3^+^; Th2: CCR6^-^CCR4^+^CXCR3^-^; Th1/17: CCR6^+^CCR4^-^CXCR3^+^; and Th17: CCR6^+^CCR4^+^CXCR3^-^) as well as the cytotoxicity marker CD226. Stained PBMCs were sorted in the La Jolla Institute (LJI) Flow Cytometry Core Facility on a FACSAria Fusion sorter.

### RNA-seq library preparation

Sequencing libraries were prepared using a low input RNA-seq prepared according to the Smart-seq2 method [22] with some modifications. 5000–15,000 PBMCs (pre-sort) or FACS isolated cell populations were lysed in TRIzol and RNA extracted using Direct-zol RNA Microprep (Zymo) with on-column DNAseI treatment. 10 μL purified RNA was mixed with 5.5 μL of SMARTScribe 5X First-Strand Buffer (Clontech), 1 μL polyT-RT primer (2.5 μM, 5’-AAGCAGTGGTATCAACGCAGAGTAC(T30)VN, 0.5 μL SUPERase-IN (Ambion), 4 μL dNTP mix (10 mM, Invitrogen), 0.5 μL DTT (20 mM, Clontech) and 2 μL Betaine solution (5 M, Sigma), incubated 50°C 3 min. 3.9 μL of first strand mix, containing 0.2 μL 1% Tween-20, 0.32 μL MgCl_2_ (500 mM), 0.88 μL Betaine solution (5 M, Sigma), 0.5 μL (5 M, Sigma) SUPERase-IN (Ambion) and 2 μL SMARTScribe Reverse Transcriptase (100 U/μL Clontech) was added and incubated one cycle 25°C 3 min., 42°C 60 min. 1.62 μL template switch (TS) reaction mix containing 0.8 μL biotin-TS oligo (10 μM, Biotin-5’-AAGCAGTGGTATCAACGCAGAGTACATrGrG+G-3’), 0.5 μL SMARTScribe Reverse Transcriptase (100 U/μL Clontech) and 0.32 μL SMARTScribe 5X First-Strand Buffer (Clontech) was added, then incubated at 50°C 2 min., 42°C 80 min., 70°C 10 min. 14.8 μL second strand synthesis, pre-amplification mix containing 1 μL pre-amp oligo (10 μM, 5’AAGCAGTGGTATCAACGCAGAGT-3’), 8.8 μL KAPA HiFi Fidelity Buffer (5X, KAPA Biosystems), 3.5 μL dNTP mix (10 mM, Invitrogen) and 1.5 μL KAPA HiFi HotStart DNA Polymerase (1U/μL, KAPA Biosystems), was added, then amplified by PCR: 95°C 3 min., 5 cycles 98°C 20 sec, 67°C 15 sec and 72°C 6 min, final extension 72°C 5 min. The synthesized dsDNA was purified using Sera-Mag Speedbeads (Thermo Fisher Scientific) with final 8.4% PEG8000, 1.1M NaCl, then eluted with 13 μL UltraPure water (Invitrogen). The product was quantified by Qubit dsDNA High Sensitivity Assay Kit (Invitrogen) and libraries were prepared using the Nextera DNA Sample Preparation kit (Illumina). Tagmentation mix containing 11 μL 2X Tagment DNA Buffer and 1 μL Tagment DNA Enzyme was added to 10 μL purified DNA, then incubated at 55°C 15 min. 6 μL Nextera Resuspension Buffer (Illumina) was added and incubated at room temperature for 5 min. Tagmented DNA was purified using Sera-Mag Speedbeads (Thermo Fisher Scientific) with final 7.8% PEG8000, 0.98M NaCl, then eluted with 25 µL UltraPure water (Invitrogen). Final enrichment amplification was performed with Nextera primers, adding 1 μL Index 1 primers (100 μM, N7xx), 1 μL Index 2 primers (100 μM, N5xx) and 27 μL NEBNext High-Fidelity 2X PCR Master Mix (New England BioLabs), then amplified by PCR: 72°C 5 min., 98°C 30 sec., 6–12 cycles 98°C 10 seconds, 63°C 30 sec., and 72°C 1 min. Libraries were size selected, quantified using the Qubit dsDNA HS Assay Kit (Thermo Fisher Scientific), pooled and sequenced on a Hi-Seq 2000 sequencer using single-end 50bp reads at a depth of 25 to 30 million single end reads per sample.

### Assay for transposase-accessible chromatin-sequencing (ATAC-seq)

50,000 FACS isolated classical monocytes or NK cells were lysed in 50 μl lysis buffer (10 mM Tris-HCl ph 7.5, 10 mM NaCl, 3 mM MgCl2, 0.1% IGEPAL, CA-630, in water) on ice and nuclei were pelleted by centrifugation at 500 RCF for 10 min. Nuclei were then resuspended in 50 μl transposase reaction mix (1x Tagment DNA buffer (Illumina 15027866), 2.5 μl Tagment DNA enzyme I (Illumina 15027865), in water) and incubated at 37°C for 30 min on a PCR cycler. DNA was then purified with Zymo ChIP DNA concentrator columns (Zymo Research D5205) and eluted with 10 μl of elution buffer. DNA was then amplified with PCR mix (1.25 μM Nextera primer 1, 1.25 μM Nextera index primer 2-bar code, 0.6x SYBR Green I (Life Technologies, S7563), 1x NEBNext High-Fidelity 2x PCR MasterMix, (NEBM0541)) for 8–12 cycles, size selected for fragments (160–290 bp) by gel extraction (10% TBE gels, Life Technologies EC62752BOX) and single-end sequenced for 51 cycles on a HiSeq 4000 or NextSeq 500.

### Sequencing analysis

RNA-seq reads were aligned to the GRCh38/hg38 assembly of the human genome using STAR (version 2.5.2a) using default parameters [23]. Gene expression values were calculated as fragments per kilobase per million mapped reads (FPKM) across GENCODE transcript exons (release 24) [24] using HOMER [25]. To remove possible contamination from genomic DNA in the RNA-seq samples, FPKM measurements were calculated for long introns (>10 kb) and the median intron FPKM per experiment was subtracted from each exon FPKM values to remove background signal. Gene expression FPKM values across all samples set to a minimum of zero and then quantile normalized. Only GENCODE transcripts with length greater than 300 bp were considered. Log2 fold change ratios were calculated using a pseudo count by adding a FPKM of 4 to both numerator (i.e. day 3, 6, 17) and denominator (i.e. day 48/convalescent) to reduce the impact of low expression noise and contamination on the lists of regulated genes. Functional enrichment was performed using HOMER using pathway definitions from Gene Ontology and HALLMARK pathways from MSigDB [26]. Promoter known motif enrichment was calculated using HOMER using sequence from -300 bp to +50 bp relative to annotated transcription start sites. Hierarchical clustering of correlated gene expression profiles, motif enrichment, and GO/pathway function enrichment values were performed using Cluster 3.0 [27] and visualized using Java TreeView [28]. For ATAC-seq, fastq files were trimmed and aligned to hg38 using bowtie2. Reads mapping to Mitochondrial DNA were removed and PCR duplicates were removed. Peaks were called using a standardized peak size using HOMER (300 bp). To compare classical monocytes and NK cells the appropriate peak files were merged and differential peaks identified using getDifferentialPeaks command (HOMER) with threshold of fold change >3 and P-value < 0.001. Motif analysis was performed on differential peak files using findMotifsGenome.pl (HOMER). All human RNA-seq and ATAC-seq data described in this manuscript are available at the National Center for Biotechnology Information (NCBI) Gene Expression Omnibus (GEO) accession number GSE123541.

### Comparison to published microarray data

Affymetrix gene expression microarray CEL files were downloaded from NCBI GEO for longitudinal DENV infection in humans (GSE43777) and ZIKV infection in Rhesus macaques (GSE93861) and processed into gene expression values using R/Bioconductor using GCRMA with default options. For the human DENV infection data, only samples performed on whole genome HG-U133plus2 microarrays were used for the comparison. Samples for the human DENV study were identified based on their annotated number of days since initial fever (G1, G2, etc.) and averaged to generate per day expression values. Rhesus macaque ZIKV infection gene expression values were averaged based on the day post infection, and human orthologues were assigned using one-to-one orthologues defined by ENSEMBL BIOMART (https://www.ensembl.org/biomart). For each study, log_2_ activation ratios were calculated using the average expression for each day compared to the average of the convalescent samples (human) or pre-infection samples (Rhesus). Microarray and RNA-seq activation ratios were compared by linking the datasets using gene symbols, using data from the highest expressed isoform in the cases where multiple isoforms exist per gene.

### Serum neutralization assay

Flow cytometry-based neutralization assay was used to evaluate SD001 serum neutralization of ZIKV (strains FSS13025 and SD001 [29]) and DENV (DENV1 strain West pacific 74 and DENV4 strain TVP-360) *in vitro*. 2×10^4^ FFU DENV or ZIKV were incubated with or without serial 3-fold dilutions (starting at 1:10) of heat-inactivated SD001 serum in 96-well round bottom plates for 1-hour at 37°C. U937 cells stably expressing DC-SIGN (1x10^5^) were seeded in each well and incubated for 2 h at 37°C with occasional rocking. After incubation, the plates were centrifuged for 5 minutes at 1500 rpm, supernatants aspirated and fresh medium added followed by incubation for 16 h at 37°C. U937 cells were then fixed, permeabilized, stained with anti-CD209 PE and 4G2 FITC (to detect ZIKV) or 2H2 FITC (to detect DENV) and analyzed using an LSRII. Percent inhibition was calculated by determining the relative infection in virus incubated with serial diluted patient serum (tests) versus no serum (control). Best fit curves and neutralizing titer 50 (NT_50_) were determined using Prism 7.0 (GraphPad).

### Cytokines analysis

Serum from 8 months prior to infection (pre-infection) as well as d3, d6, d17, and d48 POS were prepared in duplicate using the Bio-Plex Pro Human Cytokine 27-plex Assay (Bio-rad #M500KCAF0Y) per manufacturers protocol and read using a Luminex machine. Cytokine concentrations were calculated from standard curves generated using references included in the kit. The following cytokines were measured FGF basic, Eotaxin, G-CSF, GM-CSF, IFN-γ, IL-1β, IL-1ra, IL-2, IL-4, IL-5, IL-6, IL-7, IL-8, IL-9, IL-10, IL-12 (p70), IL-13, IL-15, IL-17, IP-10, MCP-1 (MCAF), MIP-1α, MIP-1β, PDGF-BB, RANTES, TNF-α, and VEGF.

## Results

**Fig 1:**
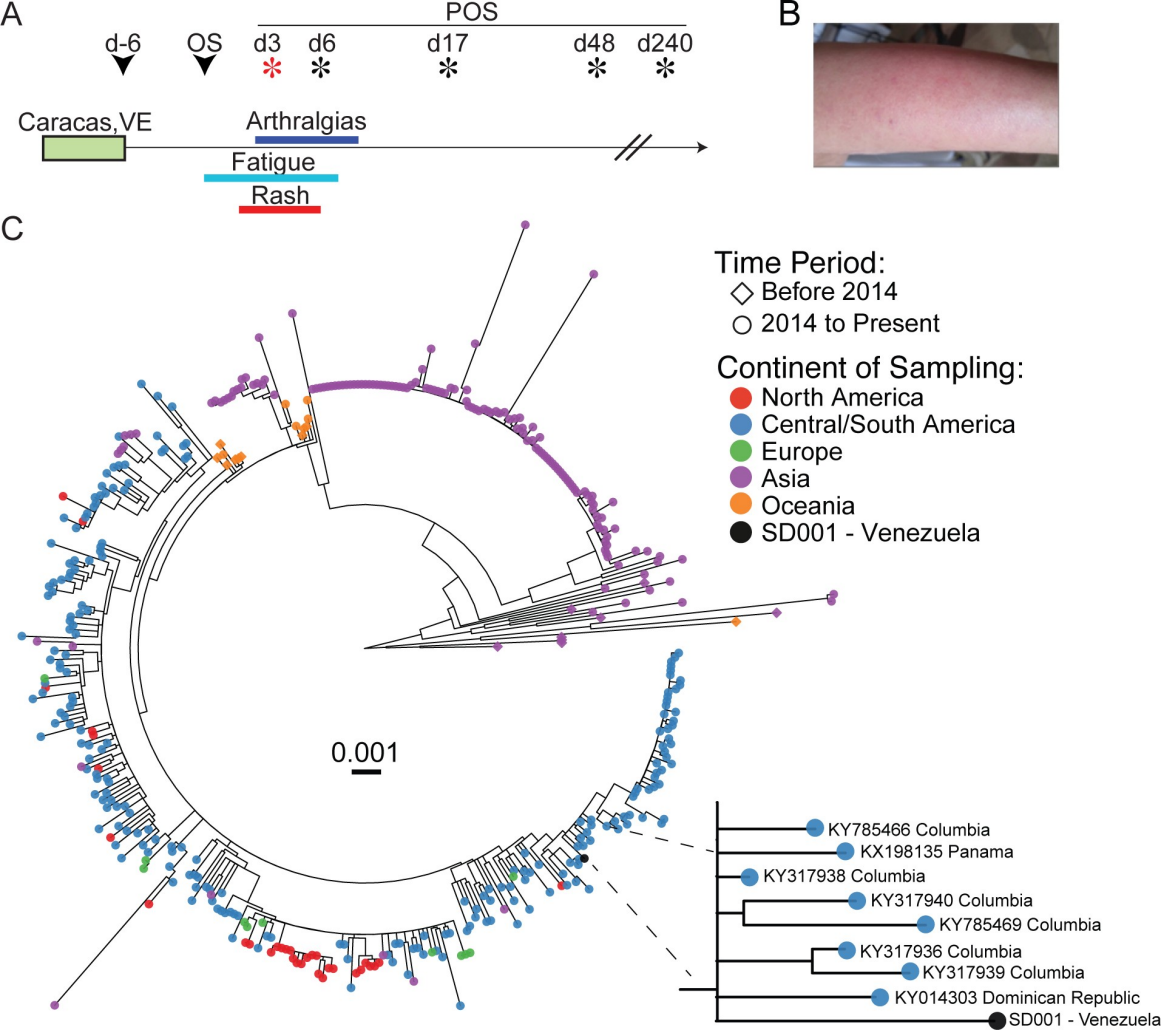
Clinical time course and sample collection. (A) Time line depicting the clinical presentation, duration of symptoms and timing of sample collection. Stars indicate days when blood samples were collected with the red star depicting the day of presentation. (B) Photograph of the left arm demonstrating the erythematous macular rash. (C) Approximate maximum likelihood phylogenetic tree of near full length ZIKV variant combined with 435 publicly available ZIKV sequences from NCBI viral genomes resource [20]. GenBank accession number are available (S1 Table). Tips are colored according to the sample sequence location. A set of 435 near full length sequence (9,038 bp) from the North America (in red), Central/South America (in blue), Europe (in green), Asia (in purple) and Oceania (in orange) were combined with the sample ZIKV SD001 (in black) and the phylogeny (midpoint rooted) was obtained using FastTree [32]. Scale bar (substitution/site) is indicated in the center. The tree topology shows the sample SD001 intermingled with sequences originating from Central/South America. A closeup of ZIKV SD001 and its nearest neighbors with GenBank accession numbers and country of origin are shown.

A middle-aged, previously healthy, dengue virus (DENV)-experienced woman developed fatigue, an erythematous pruritic macular rash, and arthralgias six days after traveling to Caracas, Venezuela in March 2016 (Fig 1A and 1B). She presented on day 3 (d3) post-onset of symptoms (POS). A comprehensive metabolic panel and complete blood count were within normal limits except for slight elevations in ALT (50 U/L, normal range 0–41 U/L) and AST (44 U/L, normal range 0–40 U/L). Serologic testing was consistent with acute flaviviral infection but did not differentiate between DENV and ZIKV infection (Table 1) [30]. A research-use nucleic acid amplification test (NAAT) (Hologic) was positive for ZIKV infection in d3 POS blood and urine samples (Table 1). Blood and urine on d3 and d6 POS were negative for DENV, as determined via qRT-PCR. Urine, from d3 and d6 POS, inoculated onto C6/36 cells produced infectious virus as measured by focus forming assay (FFA). Sequence analysis of C6/36 amplified virus was confirmed to be ZIKV using a validated population-based sequencing protocol for Zika^Br^ targeting ZIKV NS5. Phylogenetic analysis of the near complete viral genome (>9kb) showed the ZIKV San Diego isolate (ZIKV SD001 [29]) was most closely related to other Latin American ZIKV isolates downloaded from Genbank (Fig 1C) [31].

**Table 1:** Viral diagnostic test results.

	ARUP		CDC	VRDL	Hologic	
	IgG	IgM	IgM	Serum PCR	Serum NAAT	Urine NAAT
Zika	—	—	>2560 (>3)	Negative	Positive	Positive
Dengue	10.64 (>2.85)	3.55 (>2.85)	>2560 (>3)	Negative	—	—
Chikungunya	0.27 (>1.1)	0.32 (>1.1)	—	Negative	—	—

ARUP: National Reference Lab

VRDL: California Department of Public Health Viral and Rickettsial Disease Laboratory

CDC: Centers for Disease Control

—: Assay not available or not performed

NAAT: Nucleic acid amplification test

Bracketed values indicate the level at which each test is considered positive.

**Fig 2:**
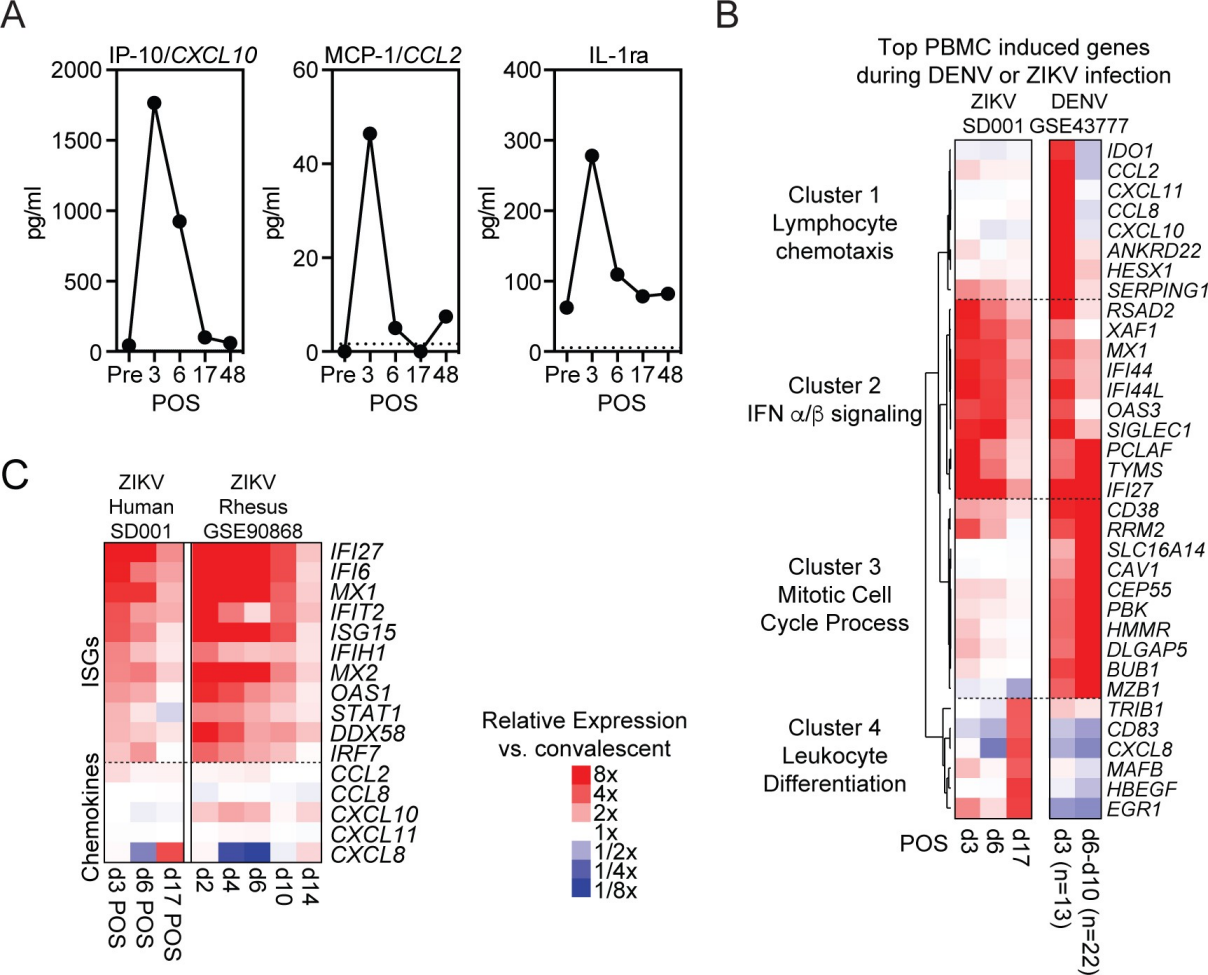
Systemic immune response to ZIKV infection. (A) Serum IP-10, MCP-1 and IL1RA levels before, during and after acute ZIKV infection. Cytokines were measured in duplicate with average shown at each time point. (B) Hierarchical clustering of the top induced genes (compared to convalescent samples) in PBMCs during acute ZIKV or acute DENV (GSE43777) infections at the indicated days post-onset of symptoms. (C) Heat map of select ISGs and cytokine genes relative expression to convalescent or baseline respectively of SD001 and rhesus macaque (GSE90868) during acute ZIKV infection [33].

To characterize the systemic immune response to ZIKV infection, we first measured circulating serum cytokine levels. Serum was collected on d3, d6, d17 and d48 POS. These samples were compared to baseline pre-infection serum collected from this individual 8 months prior to infection. We found that only a small number of cytokines, including IP-10, MCP-1 and IL-1RA, showed dramatic increases during early infection (Fig 2A). Each of these cytokines peaked on d3 POS before returning toward baseline. The levels of many inflammatory cytokines, including IFNγ and TNFα, did not change or minimally changed throughout infection (S1 Fig).

To evaluate the cellular response to infection, we first performed RNA sequencing (RNA-seq) on PBMCs. To identify induced and repressed genes during infection we compared transcriptomes at d3, d6 and d17 POS with d48 (convalescent) (Fig 2B). Hierarchical clustering of normalized PBMC transcriptional profiles showed dynamic induction patterns with strong d3 up-regulation of many interferon-stimulated genes (ISGs), which steadily declined at d6 and d17. Published human PBMC studies during acute DENV infections demonstrated sequential waves of gene expression with early induction of ISGs and inflammatory chemokines followed by a switch to induction of genes involved in cell proliferation [34]. During ZIKV infection in this individual, there was similar strong induction of type I ISGs, exemplified by *MX1*, *OAS3*, *RSAD2*, and *IFI27* genes (Cluster 2), but minimal coincident induction of chemokines involved in leukocyte chemotaxis (Cluster 1) (Fig 2B). This includes *CXCL10* and *CCL2*, that encode the chemokines IP-10 and MCP-1, that were elevated at the protein level d3 POS. Genes associated with cell differentiation and proliferation, such as *BUB1*, *DLGAP5*, *PBK* and *CEP55* (Cluster 3) were upregulated during DENV infection but not ZIKV, while *EGR1*, *HBEGF* and *MAFB* (Cluster 4), were up-regulated at d17 POS during ZIKV infection (Fig 2B).

To better understand if low-level cytokine gene induction in PBMCs was characteristic of ZIKV infection, we analyzed a published study where temporal gene expression profiles were measured in rhesus macaques following ZIKV infection [33]. PBMCs from our patient and rhesus macaques showed similar early transcriptional upregulation of ISGs but minimal chemokine gene induction with the possible exception of *CXCL10* in monkeys (Fig 2C).

**Fig 3:**
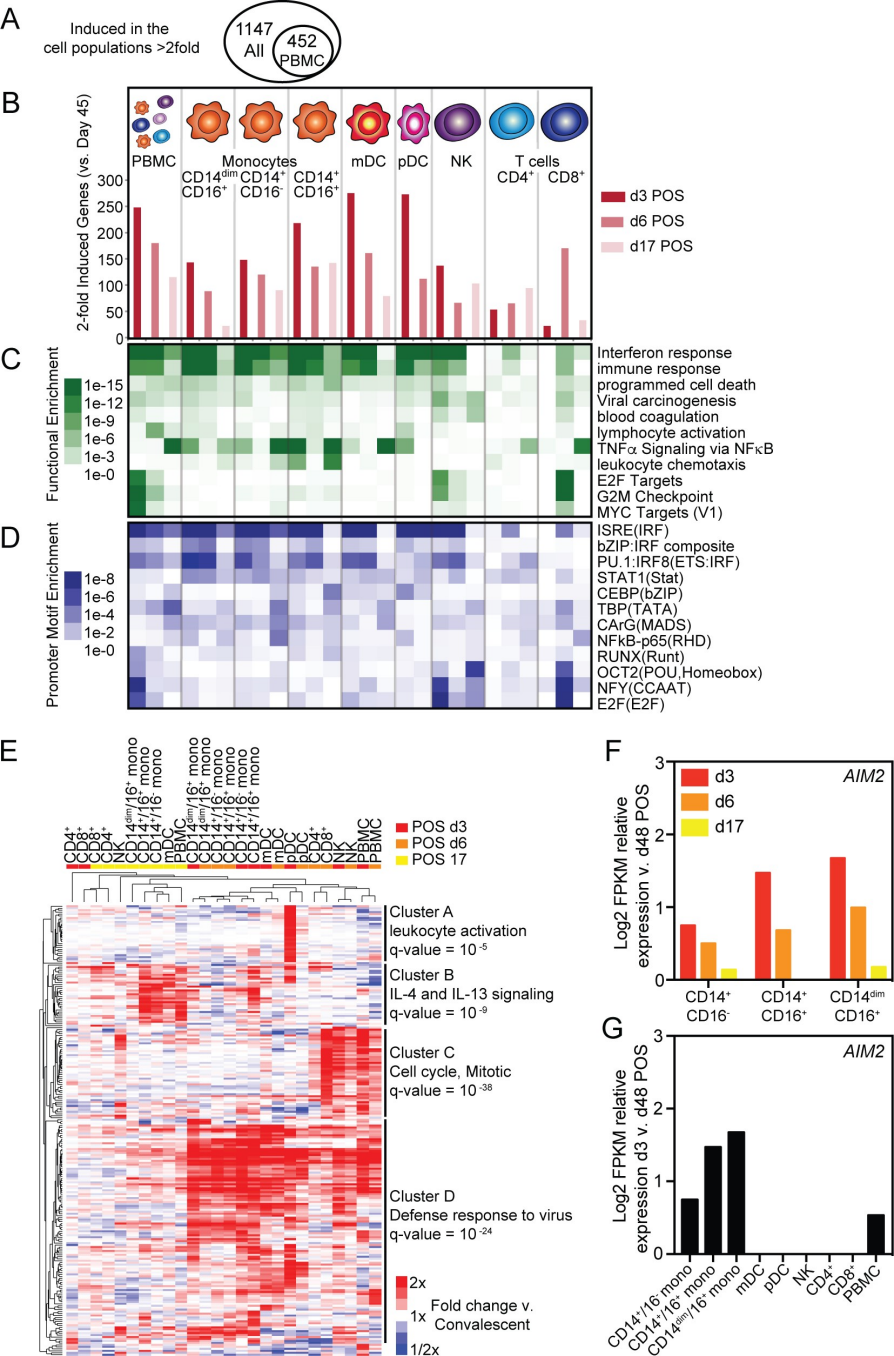
Cell-type specific and temporally regulated transcriptional response to ZIKV infection. (A) Venn diagram of the number of induced genes (at least 2-fold) determined by RNA-seq of PBMCs alone compared to analysis of PBMCs and individual cell populations. (B) Number of induced genes (at least 2-fold) at d3, d6, or d17 relative to d48 POS in specified cell populations. Corresponding (C) functional and (D) promoter motif enrichments associated with induced genes at each time point. (E) Hierarchical clustering of top induced genes in each cell type at each time point relative to d48 POS. (F) Relative Log_2_ transformed FPKM RNA-seq counts for *AIM2* in monocyte populations at indicated time points compared to d48 POS. (G) Relative Log_2_ transformed FPKM RNA-seq counts for *AIM2* in denoted cell populations at d3 compared to d48 POS.

Analyzing PBMC transcription and serum cytokines provides important information about global immune responses but lacks cell population-level resolution. To better understand how individual cell populations responds to ZIKV infection, we isolated three monocyte subsets; classical, intermediate, and non-classical; natural killer (NK) cells; two dendritic cell (DC) subsets; myeloid DCs (mDCs) and plasmacytoid DCs (pDCs); as well as CD4^+^ and CD8^+^ T cells at d3, d6, d17 and d48 POS using Fluorescence-activated cell sorting (FACS) (S2 Fig). RNA-seq transcriptional analysis of individual cell types and PBMCs together identified 1,147 genes induced at least 2-fold at d3, d6, or d17 when compared to d48 (Fig 3A). A similar analysis of PBMCs alone identified only 452 induced genes (Fig 3A). Innate immune cells (monocytes and DCs) induced the highest number of genes on d3 POS (Fig 3B). Genes up-regulated in innate immune subsets were most enriched for functional annotations associated with interferon (IFN) and immune responses at d3 and d6 POS (Fig 3C). Additionally, the promoters of genes induced at d3 and d6 POS in innate immune cells were most significantly enriched for ISRE, IRF-composite and STAT1 motifs (Fig 3D). Together, this data is consistent with early activation of type I IFN responses in innate immune populations through activation of interferon regulatory factors (IRFs) and interferon-stimulated gene factor 3 (ISGF3) transcription factors [35, 36]. In contrast to innate immune cells, the peak of T cell gene up-regulation was delayed (Fig 3B). Genes induced in CD8^+^ T cells were functionally enriched for terms associated with cell cycle progression such as E2F and MYC targets and G2M checkpoint (Fig 3C). Additionally, the promoters of these induced genes were enriched for E2F, NFY and POU binding motifs where transcription factors involved in controlling cell cycle and cell differentiation can bind (Fig 3D). Like innate immune populations, NK cells responded rapidly to infection by inducing IFN pathways (Fig 3C). However, NK cells also activated cell cycle progression pathways like CD8^+^ T cells but did so earlier, d3 compared to d6 POS, during infection (Fig 3C and 3D). Although PBMC analysis identified fewer induced genes, PBMC functional and promoter motif enrichment analyses captured many core components observed in both individual innate immune and T cell analyses (Fig 3C and 3D).

An unbiased analysis of gene expression profiles using hierarchical clustering of the top induced genes in all individual cell types and PBMCs revealed both temporal and cell type specific patterns of gene expression (Fig 3E). Gene expression at early time points, d3 and d6 POS, generally cluster together and apart from d17 responses (Fig 3E). The exception is T cells, where only d6 POS gene expression cluster in the early group. The genes driving this difference are largely induced in a time dependent manner, with anti-viral genes (Cluster B) being up-regulated early and other immune pathway genes (Cluster D) later in infection (Fig 3E). Additionally, at d3 and d6 POS, transcriptional responses cluster by cell type suggesting early transcriptional responses are in part cell type specific (Fig 3E). Population-specific induction of genes is evident from genes that are up-regulated exclusively in pDCs (Cluster A) or NK and T cells (Cluster C, Fig 3E). In contrast to all other cell types, classical and intermediate monocytes cluster together based on day POS suggesting that gene induction in these two cell types is more dependent on time POS than cell type. Many genes, including *AIM2*, an ISG involved in inflammasome activation in macrophages, is induced in both time and cell-type specific manners (Fig 3F and 3G). Additionally, *CXCL10*, *CCL2*, and *IL1RN* that encode the cytokines, IP-10, MCP-1 and IL1RA, were upregulated at least 2-fold in certain monocyte populations at d3 POS even though they were not significantly induced in PBMCs as a whole (S3 Fig).

**Fig 4:**
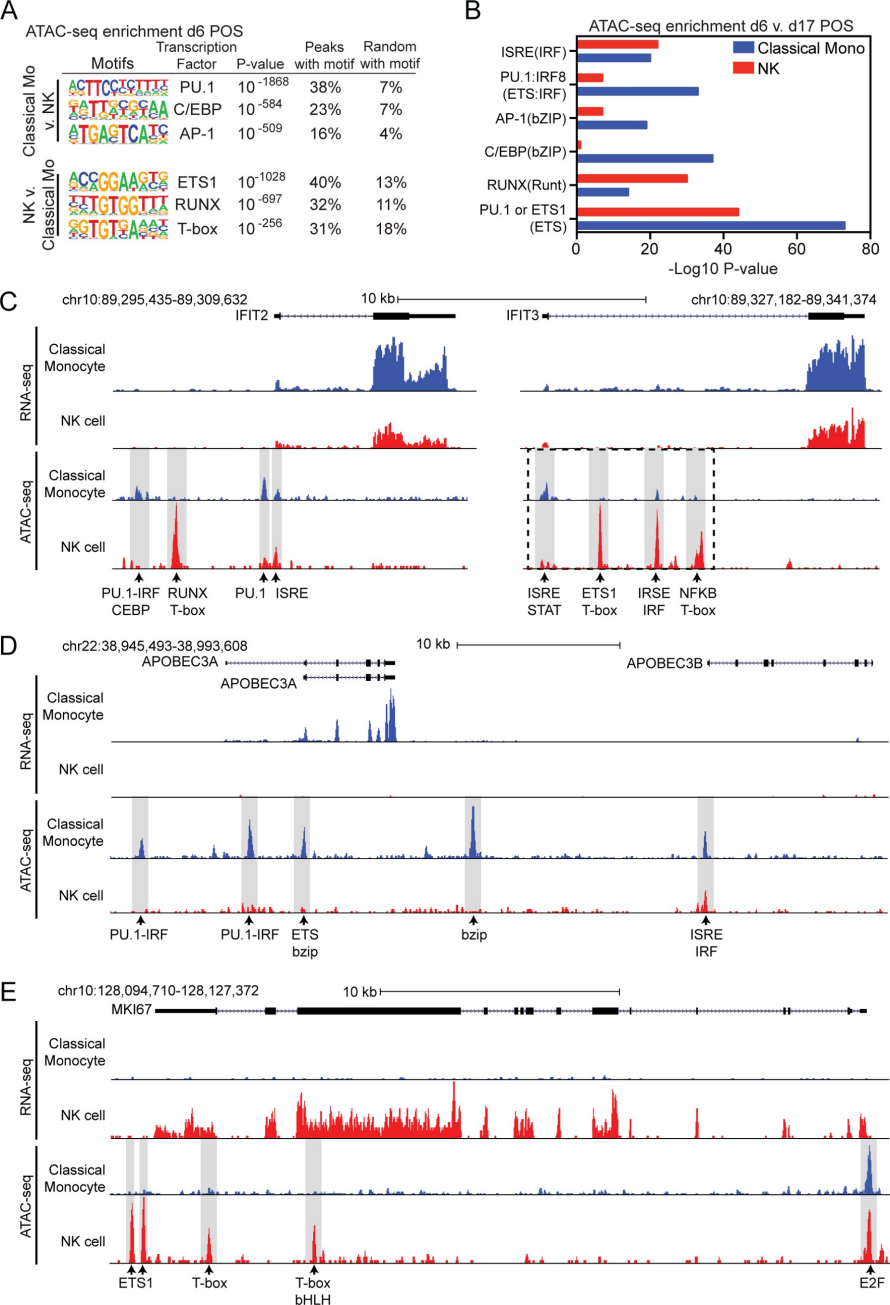
Cell-type specific ATAC-seq and gene regulation. (A) *De novo* motif enrichment at regions of open chromatin as defined by ATAC-seq in classical monocytes compared to NK cells at d6 POS. (B) Comparative motif enrichment of ATAC-seq peaks upregulated (Fold change > 3 and P-value < 0.001) at d6 compared to d17 POS in classical monocytes (blue) or NK cells (red). (C-E) UCSC browser visualization of RNA-seq (first panel) and ATAC-seq (second panel) near (C) *IFIT2 and IFIT3*, (D) *APOBEC3A* and (E) *MKI67* gene loci in classical monocytes (blue) and NK cells (red). Potential transcription factor binding motifs associated with ATAC-seq peaks are denoted. Dashed box in IFIT3 denotes area shown in S4 Fig.

Chromatin accessibility is a major component of genome regulation. Open regions of chromatin are putatively associated with genomic regulatory regions, including both promoters and enhancers. The Assay for Transposase Accessible Chromatin using sequencing (ATAC-seq) can be used to identify transcription factors (TFs) involved in regulating important functions, such as differentiation and gene regulation through the analysis of open chromatin. We did not obtain high quality d3 ATAC-seq data. However, high quality ATAC-seq data were produced using samples from d6 and d17 POS. Comparing ATAC-seq peaks in classical monocytes with NK cells on d6 POS we identified 13,792 and 13,200 peaks unique to classical monocytes and NK cells respectively. *De novo* motif analysis of these peaks identified PU.1, CEBP and AP-1 in monocytes and ETS1, RUNX and T-box in NK cells as the most enriched TF binding motifs (Fig 4A). Each of these TFs have been identified as important lineage-determining transcription factors (LDTFs) in monocytes and NK cells, respectively. To investigate TFs that may be important during the cellular response to infection we examined dynamic changes in chromatin accessibility over time. We identified 1,493 and 1,261 ATAC-seq peaks that were significantly upregulated at d6 POS compared to d17 POS in classical monocytes or NK cells respectively. In addition to cell type specific LDTFs in monocytes and NK cells, motif analysis of upregulated ATAC-seq peaks at d6 POS demonstrated increased enrichment of PU.1:IRF8 and bZIP TF binding motifs in classical monocytes (Fig 4B). In contrast, ISRE/IRF motifs were equally represented in regulated ATAC-seq peaks in classical monocytes and NK cells (Fig 4B).

To help illustrate how these data characterize individual gene loci, we considered the open chromatin landscape at genes with both common and cell-type specific patterns of regulation. Both classical monocytes and NK cells upregulated the ISGs *IFIT2* and *IFIT3* early in infection and ATAC-seq peaks were identified at sites containing ISRE motifs (Fig 4C). Although the ISRE associated peaks were common at these loci, the other ATAC-seq peaks were monocyte or NK specific and were associated with TF binding motifs enriched in the corresponding cell-type. This suggests that although both cell types induce *IFIT2* and *IFIT3* they may utilize cell type specific TFs to help regulate gene expression. Another ISG, *APOBEC3A*, was induced in monocytes but not in NK cells (Fig 4D). At this gene locus, the ATAC-seq peaks were all monocyte specific and were associated with monocyte-enriched TF binding motifs (Fig 4D). The gene *MKI67* encodes the protein Ki-67 and is a marker of proliferation. This gene was induced in NK cells early in infection but was never induced in classical monocytes (Fig 4E). The ATAC-seq peaks associated with this gene are NK-specific and associated with NK enriched TF binding motifs except for one common peak associated with an E2F motif (Fig 4E). These examples help illustrate how open chromatin patterns associated with cell-type specific transcription factors may play a role in defining common and cell-type specific patterns of gene expression (Fig 4D and 4E).

**Fig 5:**
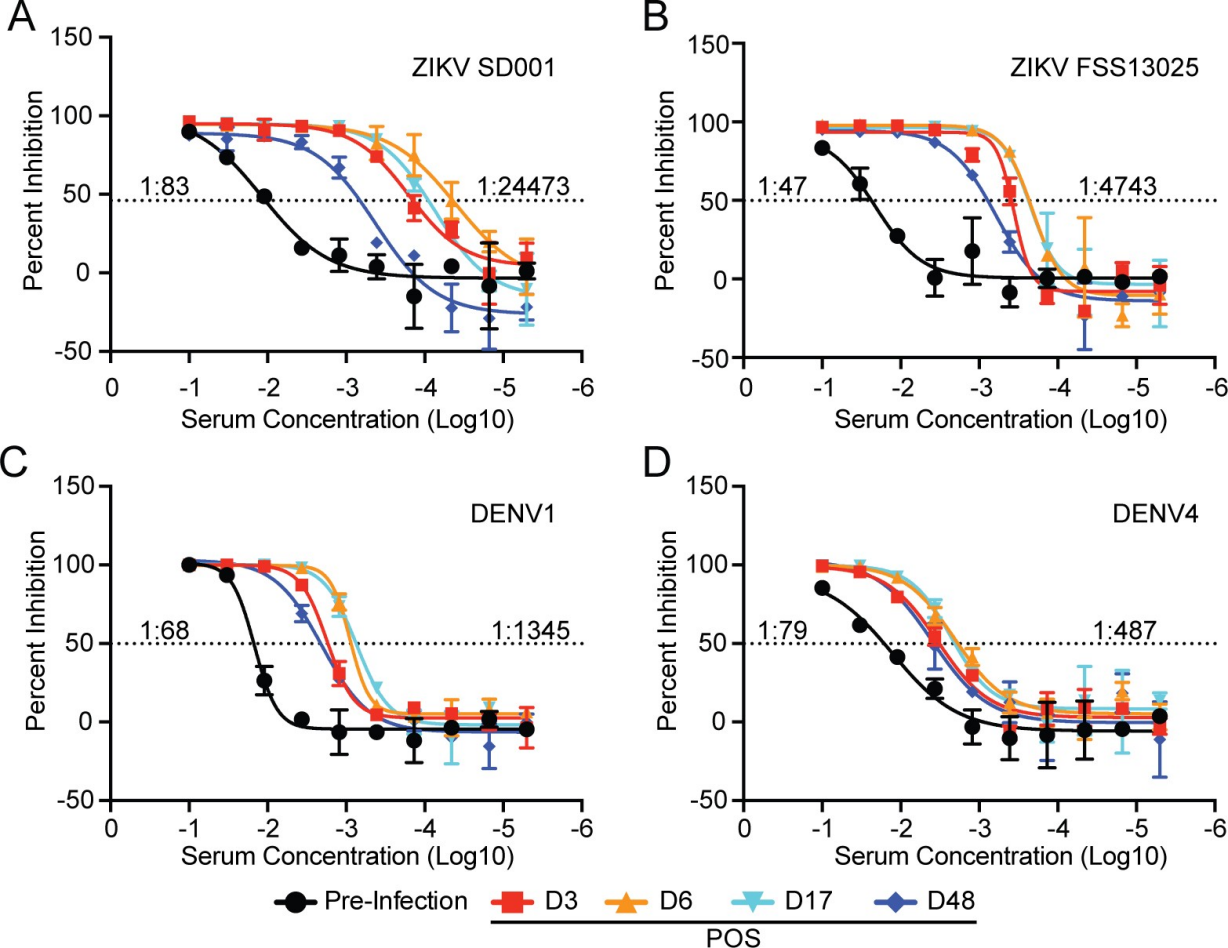
Temporal development of neutralizing Ab responses during acute ZIKV infection. (A-D) Neutralizing Ab titers against ZIKV strains (A) SD001 (B) FSS13025 and DENV strains (C) DENV1 West Pacific 74 and (D) DENV4 TVP-360 at indicated time points. Pre-infection and maximum NT_50_ titers are denoted for each virus.

We next evaluated the temporal development of adaptive immune responses. Prior to the acute ZIKV infection, this individual had low but detectable neutralizing Abs to both DENV and ZIKV strains (Fig 5A). Neutralizing Ab titers to ZIKV and DENV rapidly increased after infection, peaking on d6 POS (Fig 5A–5D). The highest neutralizing titer 50 (NT_50_) developed against the patient’s own virus followed by the related ZIKV FSS13025 (Cambodia, 2010) (Fig 5A and 5B) [37]. The NT_50_ also increased against both DENV1 and DENV4 but to a lesser degree than either ZIKV strain. These results are consistent with the idea that ZIKV infection can induce cross-reactive neutralizing Ab responses to DENV especially in individuals with prior flavivirus experience with faster kinetics relative to naïve people [17, 19].

**Fig 6:**
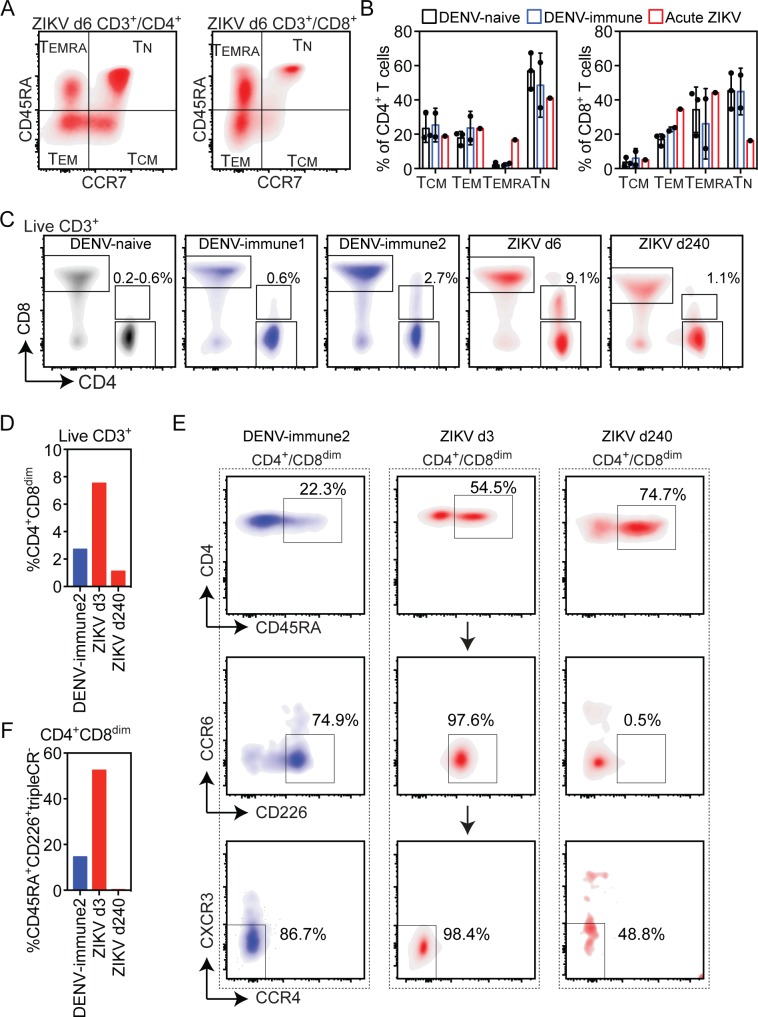
T cell immune phenotypes during acute ZIKV infection. (A) Flow cytometry analysis of CD4^+^ and CD8^+^ T cell T effector memory (T_EM_), T effector memory RA (T_EMRA_), T naïve (T_N_) and T central memory (T_CM_) populations on d6 POS. (B) Relative percentages of T cell subsets on d6 POS during ZIKV infection and in 3 DENV-naïve and 2 DENV-immune control individuals. (C) Flow cytometry analysis of live CD3^+^ cells in one representative DENV-naïve and 2 DENV-immune controls and during acute ZIKV infection d6 and d240 POS. The percentage of live CD3^+^ cells that are CD4^+^CD8^dim^ in each group is shown (D) Relative percentages of CD4^+^CD8^dim^ among live CD3^+^ cells. (E) Flow cytometry analysis of the expression of CD45RA, the cytotoxicity marker CD226, and chemokine receptors CCR6, CCR4 and CXCR3 in indicated T cell populations. (F) Percent of live CD3^+^CD4^+^CD8^dim^ T cells that are CD45RA^+^CD226^+^ and negative for CCR6, CCR4 and CXCR3 chemokine receptors.

Lastly, we assessed the T cell response by flow cytometry. Published studies have shown that the majority of DENV-specific and ZIKV-specific T cells display an effector or memory phenotype based on expression of CD45RA and CCR7 [18, 38, 39]. Moreover, in secondary DENV infections, the T cell response is associated with an expansion of T effector memory RA (T_EMRA_) and T effector memory (T_EM_) cells that can be more vigorous than in primary DENV infection [39]. Accordingly, our data on bulk populations of unstimulated T cells showed higher proportions of CD4^+^ T_EMRA_ cells and lower proportion of naïve CD8+ T cells (T_N_) at d6 POS as compared to 3 healthy DENV-naïve and 2 DENV-immune control individuals (Fig 6A and 6B). We also examined CD4 T helper (Th) subsets based on the expression of chemokine receptors (Th1: CCR6^-^CCR4^-^CXCR3^+^; Th2: CCR6^-^CCR4^+^CXCR3^-^; Th1/17: CCR6^+^CCR4^-^CXCR3^+^; and Th17: CCR6^+^CCR4^+^CXCR3^-^). No specific Th profile was observed in this individual (S2 Table), consistent with the published observation that the majority of DENV-specific CD4^+^ T cells are not associated with common Th subsets [39].

Studies of DENV-infected individuals have suggested that expanded CD4^+^ T_EMRA_ cells can exhibit a virus-specific cytotoxic phenotype that has been associated with protection against severe DENV disease [39, 40]. Cytotoxic CD4 T cells are CD45RA^+^CCR7^-^ (T_EMRA_) with increased expression of CD8α, cytotoxic effector molecules such as granzyme B and perforin, and CD226, a co-stimulatory molecule that enhances CD8 effector and cytotoxic functions. A CD4^+^ T cell population with low level CD8 expression (CD4^+^CD8^dim^) was detected in our individual with acute ZIKV patient (Fig 6C). The frequency of this population was between 3.9 and 9.1% of all CD3^+^ T cells on d3, d6, d17 and d48 POS but decreased to 1.1% by day d240 (Fig 6C and 6D). The CD4^+^CD8^dim^ population was less than 0.6% in three ZIKV-naïve controls (Fig 6C). In two DENV-immune individuals this population was 0.6% and 2.7% of all T cells (Fig 6C and 6D). At d3 POS 52.4% of CD4^+^CD8^dim^ cells were also CD45RA^+^CD226^+^ and negative for three chemokine receptors (CRs) CCR6, CCR4 and CXCR3 (Fig 6E and 6F). By d240 POS the frequency of CD4^+^CD8^dim^ cells that were CD45RA^+^CD226^+^CR^-^ fell to 0.2%. In the DENV-immune control with a significant CD4^+^CD8^dim^ population, 14.5% of this population was CD45RA^+^CD226^+^CR^-^ (Fig 6E and 6F). Based on these markers, the CD4^+^CD8^dim^CD45RA^+^CD226^+^CRs^-^ subset is likely to be cytotoxic CD4^+^ T cells.

## Discussion

Herein, we combine global PBMC and cell type-specific transcriptional and epigenetic analyses to characterize the development and resolution of an *in vivo* human immune response to an acute viral infection. This is a single patient study and therefore broad conclusions cannot be drawn. However, given the limited number and scope of published ZIKV *in vivo* response data, we feel that our study presents a unique and detailed perspective of both the innate and adaptive immune responses to ZIKV, and provides important considerations for designing future studies.

Approximately ten years prior to the ZIKV infection reported here, this individual was infected with DENV. She has no known subsequent exposure to DENV or ZIKV and has not lived in an endemic region where exposure is likely during this interval. Studies have demonstrated that prior DENV exposure influences the timing, magnitude, and quality of adaptive immune responses to ZIKV infection [18, 19]. The influence of prior DENV-exposure on innate immune responses are not understood. ZIKV is transmited by the same vector as DENV and circulates in geographical regions where DENV is endemic or hyper-endemic. Morever, ZIKV vaccine candidates have been designed for testing and deployment in DENV-endemic countries. Thus, understanding ZIKV immune responses in individuals with DENV-immunity is highly relevant.

Our analyses of PBMC and cell-specific responses demonstrate that, during acute ZIKV infection, a robust type I IFN transcriptional response was induced at early time points. Based on promoter motif analysis, this IFN response is likely driven by activation of JAK/STAT and IRF transcription factor signaling. Induction of ISG genes broadly are common to all innate immune cells tested, including monocytes (classical, intermediate and non-classical), mDCs, pDCs and NK cells. However, induction of some individual ISGs, such as *AIM2*, are induced in cell-type specific manners. Transcription analysis of bulk PBMCs is sufficient to capture a significant proportion of the response at both a pathway and gene-specific level but individual cell analysis identifies specific gene regulation and the cell type responsible for those responses during ZIKV infection that is not appreciated in the PBMC analysis. ATAC-seq enables assessment of enhancer elements distal from promoters that play important roles in modulating the immune response. This assay identified common changes in ISRE/IRF motifs in NK and classical monocytes, but also indicated substantial chromatin remodeling at sites containing cell-type specific TF binding motifs that help to explain the observed changes in gene expression. The ATAC-seq analysis was limited to d6 and d17 samples. ATAC-seq analysis at earlier time points or inclusion of other cell types could provide higher resolution of time- and cell-type dependent changes in chromatin accessibility.

Analysis of ZIKV infected cohorts in Brazil and Singapore demonstrated elevations in many serum cytokines, including IFNγ, MCP-1, IL1RA, IL-18, IL-10, IP-10 and TNFα [13, 14]. We found similar elevations in IP-10, MCP-1 and IL1RA at d3 POS but other cytokines tested showed smaller variation that is difficult to interpret. At the transcriptional level, PBMCs showed minimal induction of most chemokine genes. This included the genes *CXCL10*, *CCL2* and *IL1RN*, that encode for IP-10, MCP-1 and IL1RA. This low-level chemokine gene induction was similar to what was observed during *in vivo* ZIKV infection of macaques [33]. In contrast to PBMCs, *CXCL10*, *CCL2* and *IL1RN* were induced at least 2-fold in specific monocyte populations. Thus, specific populations, such as monocytes, or non-PBMCs may be the source of elevated IP-10, MCP-1 and IL1RA.

In our individual, neutralizing Ab titers increased rapidly post infection, peaking at d6 POS. We observed robust increases in neutralizing anti-ZIKV Ab responses with more modest increases in cross-reactive DENV-neutralization titers. This pace of neutralizing response is consistent with previous findings that humoral responses develop rapidly in DENV-immune individuals [19]. Analysis of serum from 8 months prior to infection revealed this individual had pre-existing low level neutralizing Ab titers (1:83) against ZIKV SD001 that did not prevent symptomatic ZIKV infection. Previous studies have demonstrated that individuals with remote exposure to DENV infrequently have cross-neutralizing Abs to ZIKV [41, 42]. In two studies, 0 of 19 [41] and 3 of 17 (18%) convalescent-phase [42] sera from recovered individuals with single DENV infections, had detectable cross-neutralizing Abs against ZIKV. Among persons exposed to repeat DENV infections, 3 (23%) of 13 [41] and 6 of 16 (38%) [42] convalescent-phase sera had ZIKV neutralizing Abs. Most of these individuals who develop cross-neutralizing Abs against ZIKV had relatively low Ab titers (<1:100). During DENV infections, higher levels of cross-reactive pre-infection neutralizing Ab titers in humans correlate with reduced probability of symptomatic secondary DENV infection [43]. In our individual, prior DENV exposure induced low-level ZIKV cross-neutralizing Abs that did not protect against subsequent ZIKV infection.

During our T cell phenotyping, we found a significant CD4^+^CD8^dim^ T cell subset on d3 through d48 POS that largely resolved by d240 POS. Previous studies have shown that CD4^+^CD8^dim^ T cell populations can be highly enriched for cells recognizing DENV, HCMV and HIV antigens [39, 44, 45]. In our patient, more than 50% of the CD4^+^CD8^dim^ T cells during acute ZIKV infection were CD45RA^+^CD226^+^CR^-^. This expression pattern is suggestive of cytotoxic T cells, a population not yet reported during ZIKV infection. Increased frequencies of these cells have been observed after primary and secondary DENV infections, particularly in individuals expressing HLA alleles that are associated with protection against DENV [39]. Our study provides a rationale and framework for investigating the importance of the CD4^+^CD8^dim^ T cell response in ZIKV immunity.

Collectively, these results detail the global and cell type-specific innate immune responses during an acute ZIKV infection and highlight the rapid development of neutralizing Ab and effector memory T cell responses in a DENV experienced host. These data supports accumulating evidence that prior exposure to DENV accelerates and alters adaptive immune responses likely via the presence of cross-reactive epitopes [16–19, 46]. Measuring time point- and cell type-specific transcriptional signatures of innate *vs*. adaptive immune cell populations in the blood of individuals with and without a history of flavivirus infection and vaccination can elucidate how prior flavivirus exposure might alter the magnitude, specificity, breadth, phenotype, and functionality of both humoral and cellular immune response to ZIKV. Our findings indicate that information is lost using conventional approaches and that genomic assays have the potential to provide substantial additional mechanistic insight. Combining detailed longitudinal systems biology analysis with classic immunologic techniques in future clinical studies has great potential to improve our understanding of human immune responses to pathogens at a broad level by identifying communication pathways that connect innate and adaptive immunity and regulate the balance between protection and pathogenesis. More urgently towards solving the global ZIKV and DENV problem, this approach may be invaluable in investigating the human immune response in the context of natural infection and vaccination, thereby leading to the generation of ZIKV and DENV vaccines with maximal safety and efficacy.

## Supporting information

S1 Fig Systemic cytokine responses to acute ZIKV infection. (TIF)

S2 Fig FACS strategy for separation of innate immune subsets. (TIF)

S3 Fig Cell-type specific expression of chemokine genes. (TIF)

S4 Fig Association of motifs with ATAC-seq peaks in *IFIT3*. (TIF)

S1 Table GenBank accession numbers of the ZIKV sequences from NCBI used in the viral phylogenetic analysis. (XLS)

S2 Table Frequency of CD4 T helper (Th) subsets based on the expression of chemokine receptors (Th1: CCR6-CCR4-CXCR3+; Th2: CCR6-CCR4+CXCR3-; Th1/17: CCR6+CCR4-CXCR3+; and Th17: CCR6+CCR4+CXCR3-) on specified days post onset of symptoms (POS). (XLS)
