# Supplementary figures and images for: A longitudinal systems immunologic investigation of acute Zika virus infection in an individual infected while traveling to Caracas, Venezuela
Source: PLoS Negl Trop Dis. 2018 Dec 31;12(12):e0007053. doi: 10.1371/journal.pntd.0007053 (PMC6329527; doi:10.1371/journal.pntd.0007053)

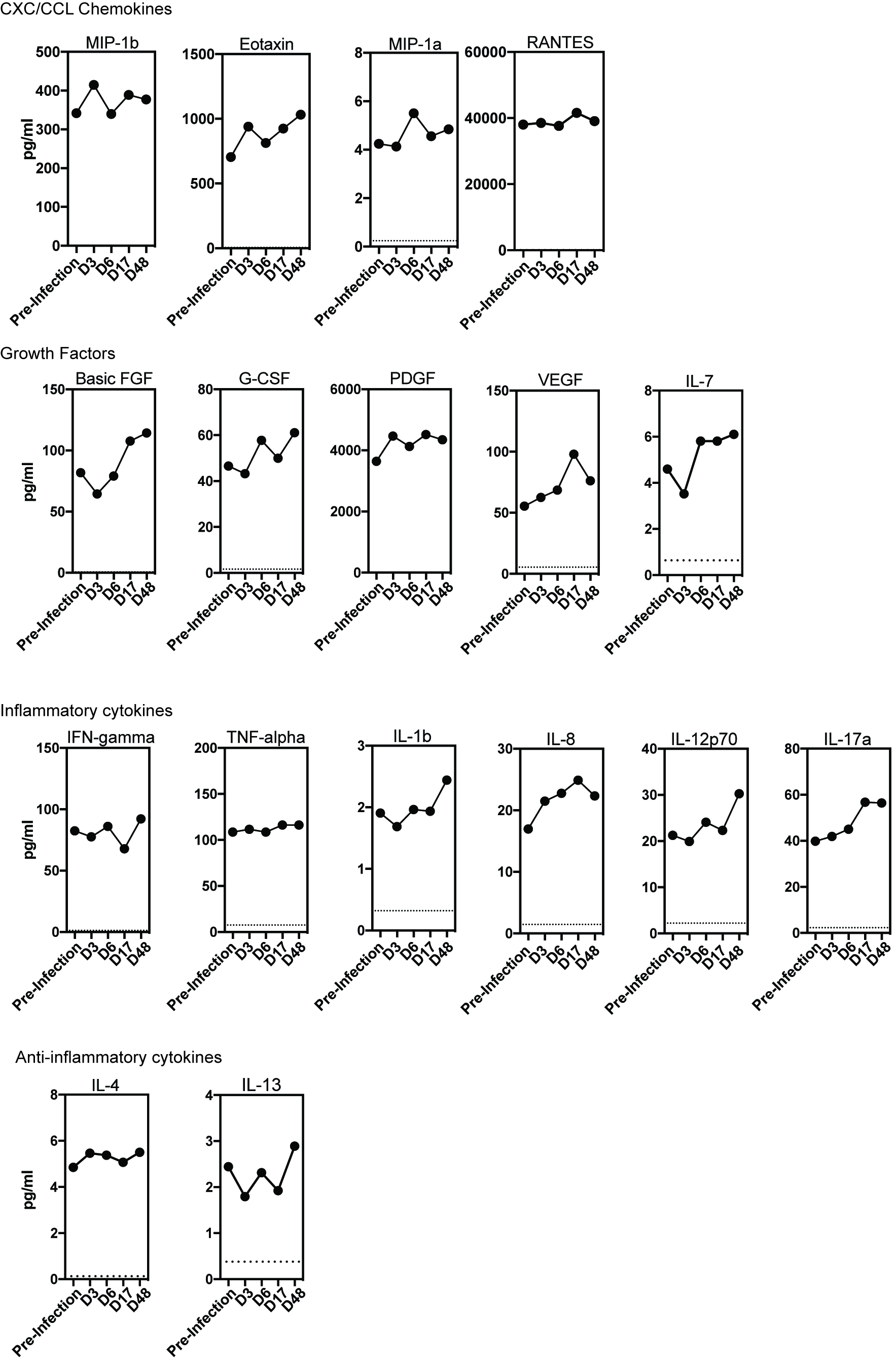

Supplement: S1 Fig — (TIF) [file pntd.0007053.s001.tif]

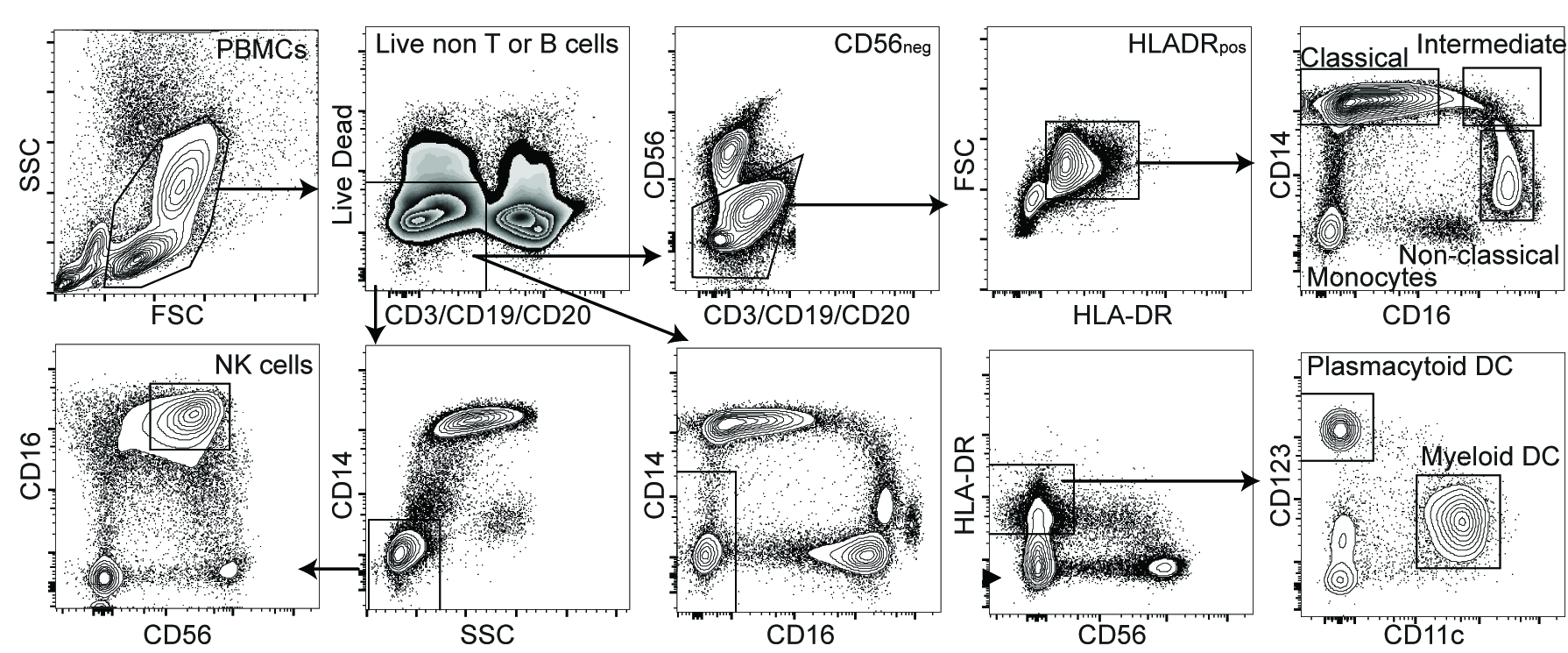

Supplement: S2 Fig — (TIF) [file pntd.0007053.s002.tif]

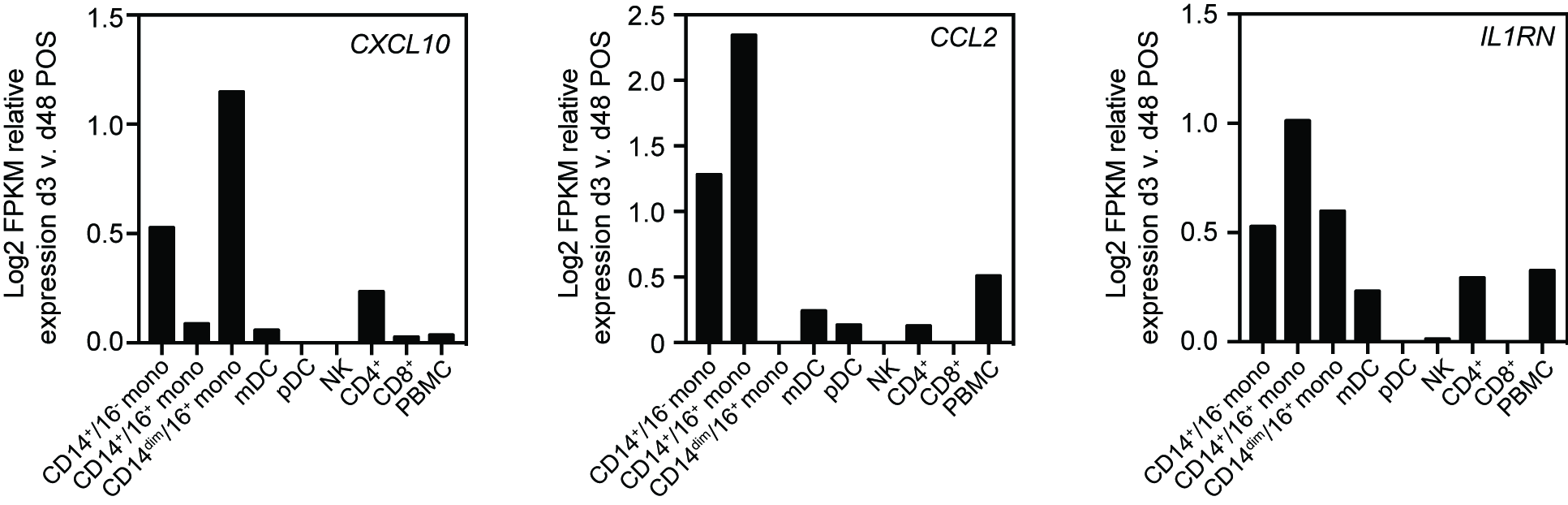

Supplement: S3 Fig — (TIF) [file pntd.0007053.s003.tif]

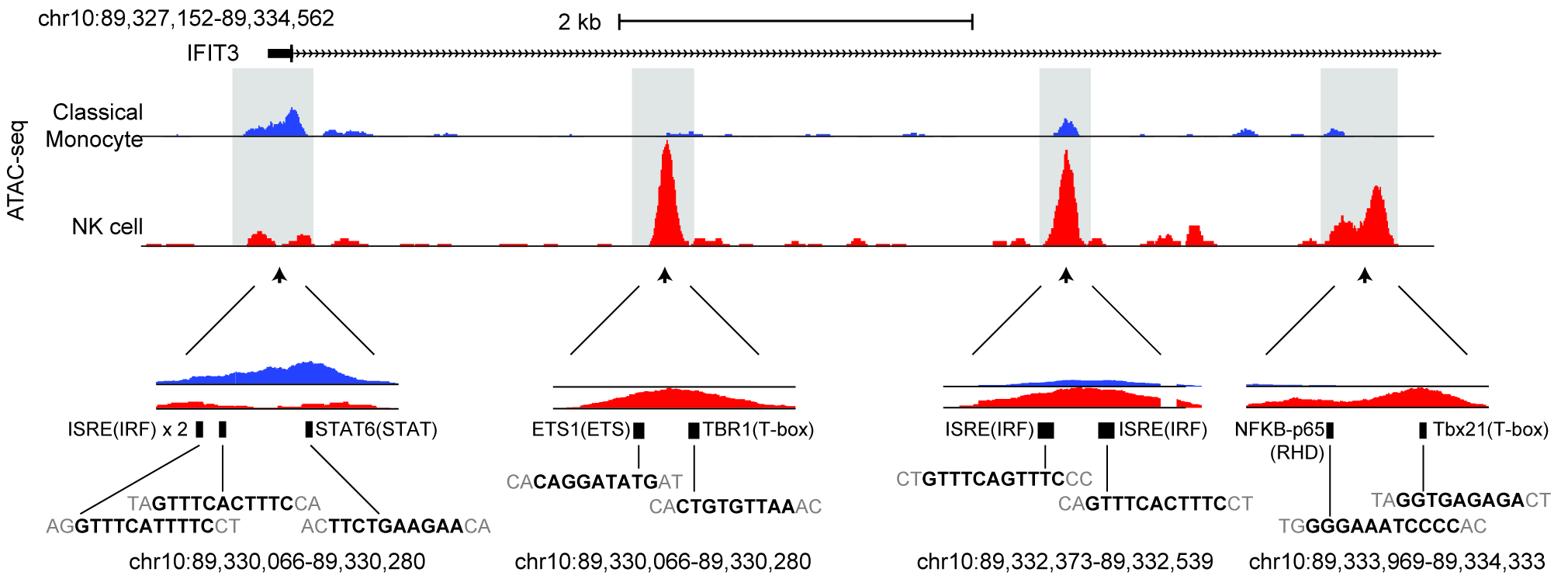

Supplement: S4 Fig — (TIF) [file pntd.0007053.s004.tif]
